# The Role of Lipotoxicity in Smoke Cardiomyopathy

**DOI:** 10.1371/journal.pone.0113739

**Published:** 2014-12-02

**Authors:** Priscila P. Santos, Fernando Oliveira, Vanessa C. M. P. Ferreira, Bertha F. Polegato, Meliza G. Roscani, Ana Angelica Fernandes, Pamela Modesto, Bruna P. M. Rafacho, Silmeia G. Zanati, Annarita Di Lorenzo, Luiz S. Matsubara, Sergio A. R. Paiva, Leonardo A. M. Zornoff, Marcos F. Minicucci, Paula S. Azevedo

**Affiliations:** 1 Internal Medicine Department, Botucatu Medical School, UNESP - Universidade Estadual Paulista, Botucatu, São Paulo, Brazil; 2 Chemistry and Biochemistry Department, Instituto de Biociências de Botucatu, UNESP - Universidade Estadual Paulista, Botucatu, São Paulo, Brazil; 3 Department of Pathology and Laboratory Medicine, Center of Vascular Biology, Weill Medical College of Cornell University, New York, New York, United States of America; University Hospital Freiburg, Germany

## Abstract

**Background/Aims:**

Experimental and clinical studies have shown the direct toxic effects of cigarette smoke (CS) on the myocardium, independent of vascular effects. However, the underlying mechanisms are not well known.

**Methods:**

Wistar rats were allocated to control (C) and cigarette smoke (CS) groups. CS rats were exposed to cigarette smoke for 2 months.

**Results:**

After that morphometric, functional and biochemical parameters were measured. The echocardiographic study showed enlargement of the left atria, increase in the left ventricular systolic volume and reduced systolic function. Within the cardiac metabolism, exposure to CS decreased beta hydroxy acyl coenzyme A dehydrogenases and citrate synthases and increased lactate dehydrogenases. Peroxisome proliferator-activated receptor alpha (PPARα) and peroxisome proliferator-activated receptor gamma coactivator 1 alpha (PGC-1α) were expressed similarly in both groups. CS increased serum lipids and myocardial triacylglycerols (TGs). These data suggest that impairment in fatty acid oxidation and the accumulation of cardiac lipids characterize lipotoxicity. CS group exhibited increased oxidative stress and decreased antioxidant defense. Finally, the myocyte cross-sectional area and active Caspase 3 were increased in the CS group.

**Conclusion:**

The cardiac remodeling that was observed in the CS exposure model may be explained by abnormalities in energy metabolism, including lipotoxicity and oxidative stress.

## Introduction

Cigarette smoke (CS) contains more than 4000 toxic substances and is responsible for the death of almost 6 million people each year [1]. Experimental and clinical studies have shown that CS induces cardiac remodeling via cardiovascular and toxic effects [2], [3], [4], [5], [6].

In previous experimental studies, rats exposed to tobacco smoke presented a different remodeling pattern associated with a drop in the systolic function [5], [7], [8], [9], [10], [11], [12]. In clinical studies, smoking has been found to be an independent risk factor for cardiac hypertrophy and dysfunction, independent of hypertension and atherosclerosis [3], [4], [13].

Indeed, the vascular damage induced by CS has been intensively studied. However, the underlying mechanisms that link the toxic effects of CS to cardiac remodeling are not well known [6].

In the setting of energy metabolism, Gvozdjakova et al. in 1984 used the term “smoke cardiomyopathy” to characterize the effects of CS on the myocardial metabolism that lead to low adenosine triphosphate (ATP) synthesis [2], [14]. Mitochondrial respiration is not only important in ATP synthesis but is also involved in reactive oxygen species (ROS) formation. Mitochondrial respiration depends on substrate oxidation, mitochondrial mass and mitochondrial function. Under normal conditions, fatty acids (FAs) are the main substrates for ATP synthesis. During heart remodeling, however, the fuel preference switches to glucose. In earlier stages of heart remodeling, this change protected the heart, in part because FAs use more oxygen when oxidized. When they are not oxidized, FAs are potential causes of lipotoxicity and ROS formation. Cardiac lipotoxicity, which is characterized by lipid storage inside the myocyte, is a potential inducer of apoptosis and dysfunction. In addition, disruptions of mitochondrial mass and function are implicated in low ATP synthesis, ROS formation and cardiac dysfunction. CS has been related to the impairment of mitochon' drial respiration; however, the substrate oxidation is poorly understood [2].

Oxidative stress plays a major role in the cardiac remodeling induced by tobacco smoke. The main sources of ROS include nicotinamide adenine dinucleotide phosphate-oxidases (NADPH oxidases) and mitochondrial respiration [15], [16]. In fact, previous studies have suggested that exposure to cigarette smoke induces NADPH oxidases [15] and affects mitochondrial respiration, increasing ROS formation [16].

ROS-induced damage is marked by lipid peroxidation of the membranes, protein damage and participation of intracellular signaling pathways [17]. Of these pathways, it is possible to highlight those that lead to hypertrophy and apoptosis. Hypertrophy has previously been described in clinical and experimental models of CS exposure [4], [11], [13]. However, apoptosis has been less studied in this model [18], [19]. In fact, apoptosis has a strict association with oxidative stress and energy metabolism. ROS-induced cardiac apoptosis is mediated through signaling systems, including intracellular calcium signaling, direct damage to the cell membrane, lipid oxidation, DNA and mitochondrial damage and proto-oncogene activation [16], [20]. The changes in energy metabolism leading to lipotoxicity are another important cause of apoptosis [21], [22], [23].

Therefore, the aim of this study was to evaluate the role of energy metabolism, including fatty acid oxidation and lipotoxicity, oxidative stress, myocyte hypertrophy and apoptosis, in the hearts of rats exposed to cigarette smoke.

## Materials and Methods

The experimental protocol was approved by the Ethics Commission on Animal Experimentation (CEEA) of our institution. It complies with the Ethical Principles of Animal Experimentation adopted by the Brazilian Board of Animal Experimentation.

Male Wistar rats weighing 200–230 g were allocated into 2 experimental groups: the control group (C), n = 8, composed of animals not exposed to cigarette smoke; and the cigarette smoke group (CS), n = 9, composed of animals exposed to cigarette smoke for 2 months.

During the first week, the smoke was released at a rate of 20 cigarettes/day (10 cigarettes twice a day in the afternoon with resting intervals of 10 minutes). The number of cigarettes was increased to a rate of 40 cigarettes/day (20 cigarettes/30 minutes in the morning and in the afternoon) until the completion of the study [24], [25]. Castardeli et al (2005) [26] observed that after one month of exposure to cigarette smoke, the smoking rats had statistically greater concentrations of carboxyhemoglobin than non smoking rats did (Control group 0.9±0.7% and Smoke group 5.3±2.8%). These carboxyhemoglobin values suggest that rats were exposed to equivalent of 20 cigarettes per day in humans [27].

### Invasive systolic blood pressure

Invasive blood pressure was measured by cannulating the femoral artery, as previously described. Invasive blood pressure was determined from the average of ten consecutive measurements of the diastolic (DBP) and systolic blood pressure (SBP), obtained through the graphic records of the polygraph. The mean blood pressure was calculated using the formula (SBP+2xDBP)/3 [28], [29].

### Echocardiographic study

All animals were weighed and evaluated via transthoracic echocardiograph exam as previously described [10]. Briefly, the exams were performed using an echocardiograph (SONO CT HDI-5000, Philips Healthcare, Netherlands, Europe) that was equipped with a 7.5 MHz phased array transducer. All measurements were obtained by the same observer according to the cutting-edge method recommended by the European Association of Echocardiography [30].

After the echocardiographic study, the animals were euthanized with a large dose of pentobarbital, their hearts were dissected, and blood was collected. Part of the heart was stored at −80°C. Transverse sections of the left ventricle were fixed in 4% buffered formalin and embedded in paraffin.

### Serum lipids

Blood samples from 6 animals in the C an CS groups were collected and serum lipids triacylglycerols (TGs), total cholesterol, low density lipoprotein (LDL) and very low density lipoprotein (VLDL), and high density lipoprotein were accessed as previously described [31], [32], [33]

### Energy metabolism, oxidative stress and cardiac triglycerides

Left ventricle (LV) samples (200 mg) from 6 animals in the C group and 5 animals in the CS group were used for the measurements of the amount of total protein [34] and lipid hydroperoxide (LH) [35] and for the enzyme determinations. Glutathione peroxidase (GSHPx, E.C.1.11.1.9), superoxide dismutase (SOD, E.C.1.15.1.1) and catalase (CAT, E.C.1.11.1.6) activity was assessed as previously specified [36], [37], [38]. The cardiac energy metabolism was assessed with 3-hydroxyacyl coenzyme-A dehydrogenase (OHADH, E.C.1.1.1.35.) and lactate dehydrogenase (LDH, E.C.1.1.1.27) and citrate synthase (CS; E.C.4.1.3.7.) activities as previously described [38], [39]. The spectrophotometric determinations were performed with a Pharmacia Biotech spectrophotometer (UV/visible Ultrospec 5000 with Swift II Application software to computer system control, 974213, Cambridge, England, UK) at 560 nm. All of the reagents were from Sigma (St. Louis, Missouri, USA).

LVs from 6 animals in each group, free of external adipose tissue, were homogenized in a mixture containing chloroform∶methanol at a ratio of 2∶1 (v/v) to extract the total lipids [40]. After 24 hours, the concentration of triacylglycerols (TGs) was determined as previously described [32].

### Western blot evaluation of Caspase 3, PPARα and PGC-1α expression

LV samples were extracted using RIPA buffer to detect Caspase 3, PPARα and PGC-1α protein expression. Samples were then centrifuged at 12.000 rpm at 4°C for 20 min, and the supernatant was collected. The supernatant protein content was quantified by the Bradford method. Samples were separated on a 10–12% SDS-polyacrylamide gel and the protein was transferred to a nitrocellulose membrane. The membrane was blocked with 5% non fat dry milk in Tris-buffered saline containing Tris 1 M, pH 8.0, NaCl 5 M and Tween 20 at room temperature for 2 hours. The membrane was then incubated with the anti-Caspase-3 rabbit monoclonal IgG (Cell SignalingThecnology, 9664), PPARα rabbit polyclonal IgG (Santa Cruz Biotechnology, Inc., Europe, sc 9000) or PGC-1α rabbit polyclonal IgG (Santa Cruz Biotechnology, Inc., Europe, sc 13067) primary antibody. The membrane was washed with Tris-buffered saline (TBS) and Tween 20 and incubated with the secondary peroxidase-conjugated antibody. Super Signal West Pico Chemiluminescent Substrate (Pierce Protein Research Products, Rockford, USA) was used to detect the bound antibodies. GAPDH (GAPDH (6C5), mouse monoclonal IgG1, Santa Cruz Biotechnology, Inc., Europe, sc 32233) was used for normalization of the blots. The nitrocellulose membranes were analyzed in the Carestream Molecular Imaging image analyzer (Carestream, Inc., USA) for a time standardized for each protein studied.

### Immunofluorescence for hypertrophy quantification

Paraffin sections from 5 animals in each group were collected on StarFrost slides and de-paraffinized by immersion in xylene and ethanol. Slides were immersed in citrate buffer pH 6.0 for antigen retrieval. The sections were blocked with bovine serum albumin and then washed in phosphate buffer saline (PBS). Wheat germ agglutinin fluorescein isothiocyanate (FITC) labeled (WGA-FITC - Sigma-Aldrich Co. LLC. L4895) 1∶40 from a 2 mg/ml stock solution was applied to the sections for 2 hours. After, the slides were washed in PBS. A liquid mountant ProLong gold antifade reagent was dropped directly onto the fluorescently labeled tissue samples. Six pictures from each section were acquired using a Leica TCS SP5 with Leica HyD confocal microscope (Leica Microsystems CMS GmbH). The average section picture contained at least 180 myocytes, and the myocyte cross-sectional area (CSA) was determined (Image-Pro Plus 3.0, Media Cybernetics, Silver Spring, MD).

### Statistics

All of the grouped data were evaluated with GraphPad Prism 5 software. Variables from each group were compared using Student's *t*-test or a Mann-Whitney test. The data were expressed as the mean ± standard deviation or the median and the percentile (25–75). P values of less than 0.05 were considered to indicate statistical significance.

## Results

At the end of the experiment, the animals in the two groups had similar body weights (C = 382±21 g; CS = 370±28 g) (*p* = 0.3) and heart rates (C = 303±47 beats per min; CS = 287±26 beats per min) (*p* = 0.4) and presented normal mean blood pressure (C = 77±8 mmHg; CS = 89±11 mmHg) (*p* = 0.1). The echocardiographic study showed enlargement of the left atria (LA) and an increase in the left ventricular systolic volume (LVSV), followed by reduced systolic function in the CS animals (Table 1).

**Table 1:** Echocardiographic data.

	C (8)	CS (9)	p
**LVDD/BW (mm/kg)**	19.0±1.67	19.5±1.41	0.552
**LVSV/BW (mm/kg)**	7.71±0.80	8.99±1.30	0.030
**DPWT/BW (mm/kg)**	3.22±0.34	3.42±0.44	0.312
**LVRWT**	0.34±0.05	0.35±0.04	0.800
**LAD/BW (mm/kg)**	10.3(10.1–10.7)	11.1(10.6–12.6)	0.038
**LAA/BW (cm^2^/kg)**	0.52 (0.47–0.60)	0.63(0.60–0.73)	0.030
**LAA/RAA**	1.12±0.13	1.33±0.19	0.020
**LVMI (g/kg)**	1.47±0.20	1.51±0.20	0.710
**EF**	93.2 (92.1–94.4)	90.1(86.7–92.9)	0.048
**FS%**	59.3 (57.1–61.9)	53.7 (49.0–58.8)	0.048
**E/A**	1.52±0.28	1.63±0.2	0.322
**EDT**	51.8±5.39	53.0±9,50	0.781
**IRTc**	58.4±13.0	51.1±7.89	0.176

LVDD: left ventricular diastolic diameter; LVSV: left ventricular systolic diameter; DPWT: diastolic posterior wall thickness; LVRWT: left ventricular relative wall thickness; LAD: left atrial diameter; LAA: left atrial area; RAA: right atrial area; LVMI: left ventricular mass index; FS: fractional shortening; EF: ejection fraction; E/A: waves E/A ratio; EDT: wave E decelerating time; IRTc: isovolumetric relaxation time corrected with cardiac frequency. The data are expressed as the mean ± standard deviation or the median (percentile 25–75). Significance level 5%.

**Figure 1:**
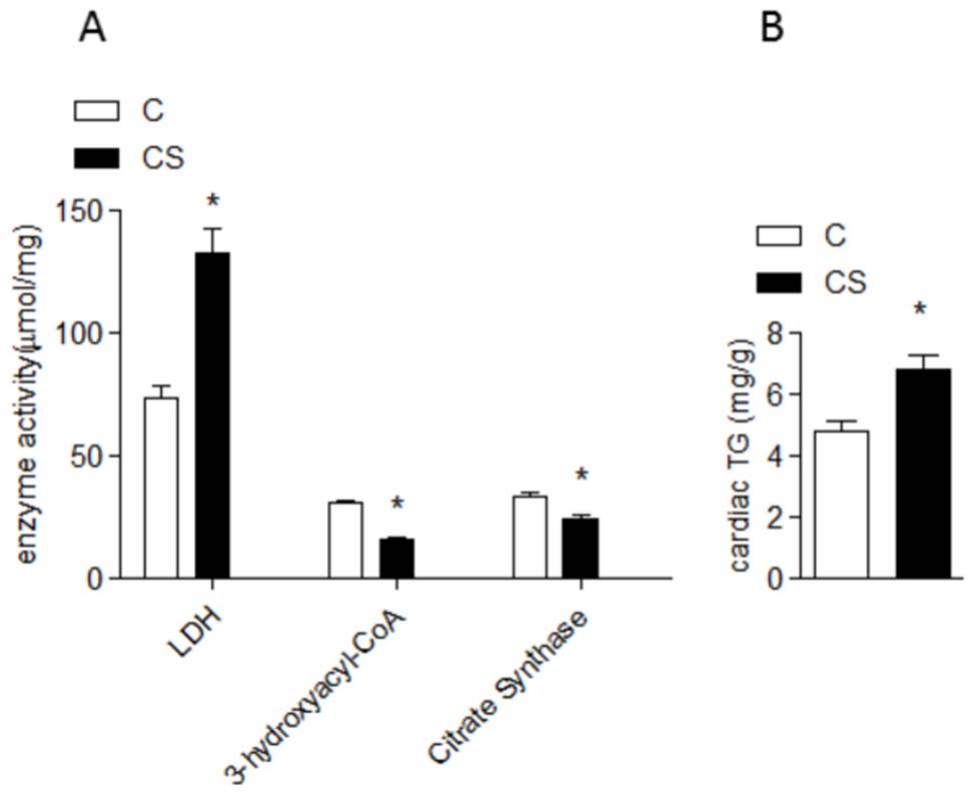
Energy Metabolism. C: control group; CS: cigarette smoke group; LDH: lactate dehydrogenases C vs. CS *p*<0.001; 3-hydroxyacyl-CoA: 3-hydroxy acyl coenzyme A dehydrogenases C vs. CS *p* = 0.004; citrate synthase C vs. CS *p* = 0.008; cardiac TG: cardiac triacylglycerol C vs. CS *p* = 0.004.

**Figure 2:**
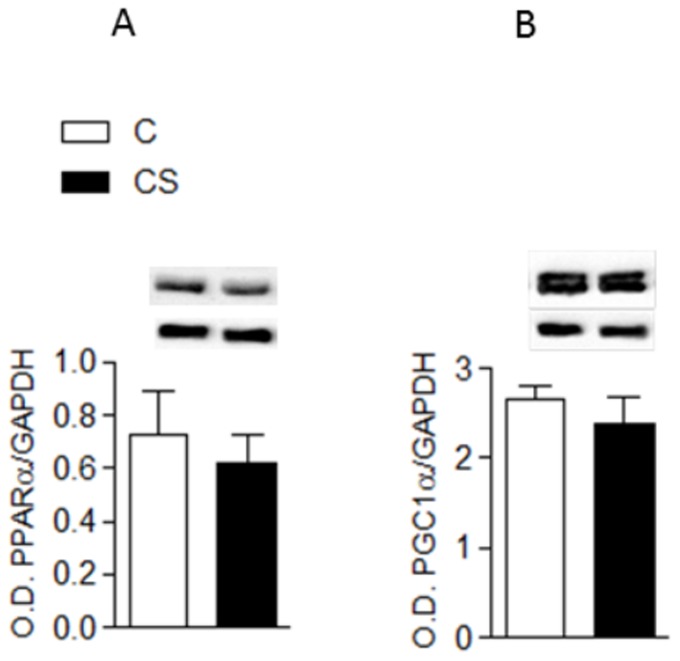
Modulators of FA oxidation and mitochondrial biogenesis. C: control group; CS: cigarette smoke group; O.D.: optical density; GAPDH: glyceraldehyde-3-phosphate dehydrogenase; MW: molecular weight. **2A**: upper bands: PPARα: peroxisome proliferator-activated receptor alpha; lower-bands: GAPDH; PPARα/GAPDH: C vs. CS *p* = 0.6. **2B**: upper bands: PGC-1α: peroxisome proliferator-activated receptor gamma coactivator 1 alpha; lower bands: GAPDH; PGC-1α/GAPDH: C vs. CS *p* = 0.5.

**Figure 3:**
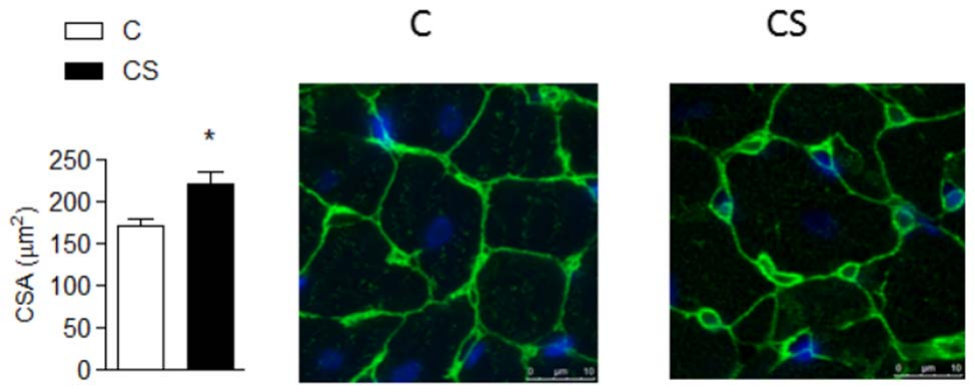
Myocyte CSA. C: control group; CS: cigarette smoke group; CSA: cross-sectional area C vs. CS *p* = 0.018.

**Figure 4:**
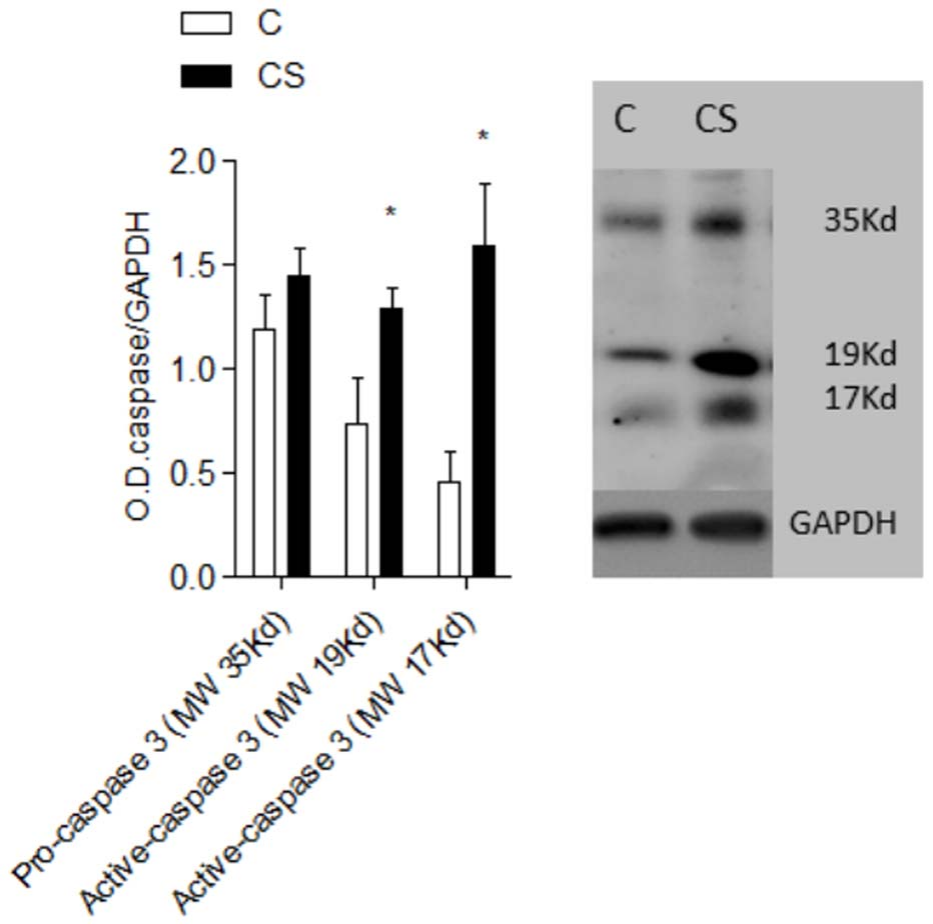
Apoptosis. C: control group; CS: cigarette smoke group; O.D.: optical density; GAPDH: glyceraldehyde-3-phosphate dehydrogenase; MW: molecular weight. Upper-bands: pro-Caspase and active Caspase subunits; lower bands: GAPDH; pro-Caspase/GAPDH C vs. CS *p* = 0.2; active-Caspase 3 (MW 19 kD) C vs. CS *p* = 0.04; active-Caspase 3 (MW 17 kD) *p* = 0.008.

Within the cardiac metabolism, exposure to cigarette smoke lowered OHADH and citrate synthases and increased LDH (Figure 1A). Serum lipid analysis showed that cigarette smoke increased TGs, total cholesterol, LDL and VLDL and decreased HDL (Table 2). In addition, CS increased myocardial TGs (Figure 1B). On the other hand, PGC-1α and PPARα were similar across the two groups (Figure 2A and B). CS rats presented with an increase in oxidative stress and a decrease in antioxidant defense (Table 3). Finally, myocyte CSA (Figure 3) and active Caspase 3 (Figure 4) were increased in the CS group.

**Table 2:** Serum lipids.

	C (6)	CS (6)	P
**TG (mg/dL)**	88.5(85.9–90.5)	150(140–162)	<0.001
**Total cholesterol (mg/dL)**	97.7(95.8–98.4)	188(160–210)	<0.001
**LDL (mg/dL)**	26.0(23.9–27.4)	122(100–146)	<0.001
**VLDL (mg/dL)**	17.7 (17.4–18.1)	30.1(28.1–32.4)	<0.001
**HDL (mg/dL)**	53.4±2.82	31.6±3.51	<0.001

TG: triglycerides; LDL: low density lipoprotein; VLDL: very low density lipoprotein; HDL: high density lipoprotein. The data are expressed as the median and the percentile 25–75 or the mean ± standard deviation. Significance level 5%.

**Table 3:** Oxidative Stress.

	C (6)	CS (5)	p
**SOD (µmol/mg)**	19.4±1.92	11.2±0.91	<0.001
**GSH-Px (µmol/mg)**	37.4±4.81	17.5±3.93	<0.001
**Catalases (µmol/mg)**	80.1±10.4	62.1±16.0	0.051
**LH (µmol/mg)**	133±14.7	175±11.5	<0.001

SOD: superoxide dismutase; GSH-PX: glutathione peroxidases; LH: lipid hydroperoxide. The data are expressed as the mean ± standard deviation. Significance level 5%.

## Discussion

The aim of our study was to evaluate the potential mechanisms involved in the remodeling process induced by exposure to cigarette tobacco, including energy metabolism, oxidative stress, myocyte hypertrophy and apoptosis. Our data suggest that alterations in energy metabolism might be a key modulator of the remodeling process induced with smoking, which is associated with apoptosis, oxidative stress and cellular growth.

The first finding of our study was that smoking increased the size of the left atrium and left ventricle, which was associated with a drop in the systolic function, independent of the blood pressure. Therefore, the data showed that the toxic effects of cigarette smoke led to the remodeling process. In experimental studies, CS has been implicated in different remodeling patterns [12], [22]. In addition, clinical studies have shown that the toxic effects of cigarette smoke lead to hypertrophy and cardiac dysfunction [3], [4]. Meanwhile, the reasons why cigarette smoke leads to the remodeling process are not well known.

The present study showed that exposure to cigarette smoke induced alterations in cardiac energy metabolism. The decrease in OHADH activity and increase in cardiac TGs represents an impairment in FA oxidation. In addition, there was an increase in LDH activity, which indicates an increase in pyruvate or lactate acid formation.

FA oxidation regulation includes the PPARα and PGC-1α pathways; however, the expression of these two factors was similar in the two groups [21], [23], [41]. One explanation for this result is that low expression and activity may not be concomitant with low FA oxidation [42]. Therefore, the role of PPARα and PGC-1α in the cardiac remodeling induced by CS remains unclear.

In addition, the low FA oxidation and TG storage inside the myocytes of CS rats reflects characteristic lipotoxicity. The lipotoxicity process has a critical role in cardiac dysfunction, though the mechanisms are not well understood. One possibility is that lipotoxicity-induced apoptosis plays a major role in cardiac dysfunction [22], [23], [41]. Importantly, in this study, CS contributed to the accumulation of lipids inside the myocytes through two possible mechanisms: (1) inhibition of OHADH via CS-induced decreased FA oxidation and (2) CS-induced increase in the total serum cholesterol, TG and VLDL, which are indirect markers of lipolysis.

In fact, the effect of cigarette smoke on serum lipids has been described previously [43]. One possible mechanism is that CS increases catecholamine levels, which further increases lipolysis and ultimately increases FA uptake by myocytes [44], [45]. Another mechanism might be an inhibition of lipoprotein lipases [44], [46].

Investigation into the mitochondrial function determined that, CS exposure decreased the activity of the citrate synthases, which are crucial enzymes in the citrate cycle. Low activity of these enzymes may represent an impairment in the mitochondrial respiration and function [21]. While PGC1-α is a key modulator of mitochondrial biogenesis, it did not participate in the CS-mediated damage [21], [22]. In a previous study, PGC1-α was observed in edema and was decreased in mitochondria crypts in rats exposed to CS [47]. Thus, alteration in mitochondrial mass and function is a potential mechanism for the impairment of ATP synthesis in CS exposed rats [14], [47].

Mitochondria are also an important source of ROS, which lead to an increase in oxidative stress. Impairment of mitochondrial respiration, increased demand of energy transference, permeability transition pores (PTPs) and FA transportation by uncoupled proteins through mitochondrial membranes all contribute to ROS generation [21], [23], [48]. In our study, FA oxidation was decreased. Non-oxidized FAs may bind to uncoupled proteins in the mitochondrial membrane, favoring ROS formation. In addition, the citric acid cycle appeared to have been compromised because citrate synthases were decreased, which compromised mitochondrial respiration, favored ROS formation and decreased ATP synthesis.

Independently of the ROS source, in this study, there was an increase in LH and a decrease in antioxidant enzymes, suggesting that oxidative stress participates in the remodeling process induced by CS. In general, ROS exerts an important role in the pathophysiology of the lesions induced by cigarette smoke [49], [50]. In cardiac tissue, oxidative stress participated in the cardiac remodeling induced by CS in normal rats and after myocardial infarction [9], [19]. Oxidative stress promotes lipid peroxidation and damage to the cell membranes, which alter the structure and function of suitable cells. Additionally, increased oxidative stress is involved in a variety of intracellular signaling pathways that take part in the remodeling process, including hypertrophy and apoptosis [17], [20], [49].

Hypertrophy and apoptosis were observed in the CS group. Although they represent different phenotypes, the intracellular signaling pathways for apoptosis and hypertrophy are similar and involve mitogen-activated protein kinase (MAPK) signaling [51]. Gu et al. showed that rats exposed to tobacco smoke exhibited activation of the MAPK signaling cascade [52]. Previous studies have revealed different features with respect to hypertrophy. In fact, rats exposed to tobacco smoke may present 4 different patterns of cardiac remodeling. Overall, the normal pattern (normal left ventricle mass index (LVMI) and normal left ventricle relative thickness) is the most common, followed by eccentric hypertrophy (increased LVMI and normal LVRT) [5]. In general, histologic analyses have shown an increase in the myocyte CSA of rats exposed to CS [10], [19]. In this study, a normal remodeling pattern was observed. However, myocyte CSA was increased in the CS group, indicating myocyte hypertrophy.

In fact, apoptosis increased in CS-exposed rats, as evidenced by the increased expression of the active forms of Caspase 3. This enzyme is the final effector of apoptosis induced by extrinsic factors [51]. Recently, Zhou et al. observed CS-induced apoptosis in cardiac tissue [18], [19]. The authors suggest that oxidative stress was the major cause of apoptosis in their study. In actuality, apoptosis has a strict association with oxidative stress and energy metabolism. Direct damage to the cell membrane and ROS-induced DNA and mitochondrial damage are all signs of ROS-induced apoptosis [16]. The present study generated evidence for increased ROS and mitochondrial dysfunction as a potential pathway for myocyte hypertrophy and apoptosis.

Importantly, the present study demonstrated that cardiac lipotoxicity is induced by CS. Therefore, lipotoxicity should be considered another key mechanism in addition to oxidative stress that can explain cardiac apoptosis and dysfunction. Indeed, additional early time-point study showed that changes in energy metabolism and the increase in myocardial TG precede the morphologic and functional heart alterations (data S1).

In conclusion, the present study determined that exposure to cigarette smoke induces abnormalities in energy metabolism, marked by low FA oxidation and mitochondrial dysfunction, which are a potential cause of ROS formation, lipotoxicity and low ATP synthesis. Therefore, these mechanisms could potentially explain smoking-induced cardiac hypertrophy, apoptosis and dysfunction. In addition, high levels of serum lipids may intensify cardiac lipotoxicity in CS-exposed rats.

## Supporting Information

Data S1 (DOCX)
