# Supplementary material for: The Role of Lipotoxicity in Smoke Cardiomyopathy
Source: PLoS One. 2014 Dec 2;9(12):e113739. doi: 10.1371/journal.pone.0113739 (PMC4252176; doi:10.1371/journal.pone.0113739)
Supplement: Data S1 — (DOCX) [file pone.0113739.s001.docx]

Data S1

We performed additional experiments to show that changes in metabolism precede changes in cardiac function. Male Wistar rats weighing 200-230 g were allocated into 2 experimental groups: the control group (C), n=6, composed of animals not exposed to cigarette smoke; and the cigarette smoke group (CS), n=6, composed from animals exposed to cigarette smoke during 1 month. After this period, the rats were submitted to Echocardiographic study. Activity of energy metabolism enzymes and concentration of triglycerides were performed on left ventricle samples.

The echocardiographic data and activity of energy metabolism enzymes and concentration of myocardial triglycerides are presented in Tables 1 and 2 respectively. The echocardiographic variables were not different between groups. However, the exposure to cigarette smoke changed the cardiac energy metabolism and increased myocardial TG. The activity of 3-hydroxyacyl-CoA and citrate synthase enzyme was lower in CS group compared to control. In addition, the activity of LDH was higher in CS than control. These results were similarly to the ones shown in the two months experiment. Therefore, the changes in energy metabolism and the increase in myocardial TG precede the morphologic and functional heart alterations. This information was added to the manuscript (Page 13 lines297-300).

**Table 1**: Echocardiographic data

|  | **C** | **CS** | **p** |
| --- | --- | --- | --- |
|  | **(6)** | **(6)** |  |
| **LVDD/BW (mm/kg)** | 18.3±0.91 | 16.9±1.77 | 0.121 |
| **LVSV/BW (mm/kg)** | 8.00±0.97 | 7.31±0.61 | 0.167 |
| **DPWT/BW (mm/kg)** | 4.45±0.27 | 4.47±0.50 | 0.932 |
| **LVRWT** | 0.49±0.04 | 0.53±0.08 | 0.247 |
| **LAA/BW (cm^2^/ kg)** | 0.55(0.55-0.56) | 0.56 (0.54-0.59) | 0.818 |
| **LAA/RAA** | 1.00 (1.00-2.00) | 1.50 (1.00-2.00) | 0.699 |
| **EF** | 92.3(91.6-93.0) | 92.3 (90.1-93.6) | 0.699 |
| **FS%** | 57.5 (56.2.0-58.8) | 57.5 (53.8-60.0) | 0.699 |
| **E/A** | 1.51±0.16 | 1.38±0.17 | 0.216 |
| **EDT** | 58.0±5.3 | 53.2±11.9 | 0.384 |
| **IRTc** | 30.0 (22.0-30.0) | 30.0 (30.0-37.0) | 0.132 |

LVDD: left ventricular diastolic diameter; LVSV: left ventricular systolic diameter; DPWT: diastolic posterior wall thickness; LVRWT: left ventricular relative wall thickness; LAD: left atrial diameter; LAA: left atrial area; RAA: right atrial area; FS: fractional shortening; EF: ejection fraction; E/A: waves E/A ratio; EDT: wave E decelerating time; IRTc: isovolumetric relaxation time corrected with cardiac frequency. The data are expressed as the mean ± standard deviation or the median (percentile 25-75). Significance level 5%.

**Table 2:** Triglycerides and enzymes of cardiac energy metabolism.

|  | **C** | **CS** | **p** |
| --- | --- | --- | --- |
|  | **(6)** | **(5)** |  |
| **LDH (µmol/mg)** | 87.9±19.0 | 124.7±25.4 | 0.009 |
| **3-hydroxyacyl-CoA (µmol/mg)** | 22.3±2.50 | 14.5±2.07 | <0.001 |
| **Citrate synthase (µmol/mg)** | 31.21±2.77 | 21.06±2.67 | <0.001 |
| **TG (µmol/mg)** | 5.15±1.66 | 8.23±1.47 | 0.007 |

LDH: lactate dehydrogenases; 3-hydroxyacyl-CoA: 3-hydroxy acyl coenzyme A dehydrogenases; citrate synthase; TG: cardiac triacylglycerol. The data are expressed as the mean ± standard deviation. Significance level 5%.
